# Stamping Lithography on Arbitrary Surfaces based on Self‐Assembly of Colloidal Particles

**DOI:** 10.1002/advs.202518380

**Published:** 2025-11-29

**Authors:** Guoxu Yu, Heyang Zhang, Yiming Li, Lele Song, Jinglin Jia, Lei Chen, Ding Weng, Yuan Ma, Jiadao Wang

**Affiliations:** ^1^ Department of Mechanical Engineering Tsinghua University Beijing 100084 P. R. China

**Keywords:** 3D circuits, micro/nano manufacturing, self‐assembly, soft lithography

## Abstract

Three‐dimensional (3D) circuits on curved surfaces have been widely used in conformal antennas, intelligent skins, metasurfaces, and bionic electronics. Traditional planar circuit technologies represented by photolithography are confronted with the problem of being difficult to apply to curved surfaces. To solve this problem, transfer printing technology uses planar stamps to transfer devices onto curved surfaces fabricated by photolithography, but faces the problem of over‐stretching when adapting to non‐developable surfaces. Meanwhile, in‐situ additive manufacturing technologies on curved surfaces, such as inkjet printing and laser direct writing, have low manufacturing efficiency, and the available materials are minimal. Herein, this paper proposes a new method called Stamping Lithography, which comprises a resist mask stamping process based on self‐assembly of colloidal particles (RSSA) and a subsequent etching process similar to those used in planar circuit technologies, making it possible to fabricate circuits using various materials on large‐area non‐developable surfaces. By fabricating circuits on hemispherical substrates, this approach demonstrates the potential of non‐developable surfaces. This work presents a practical strategy for the fabrication of 3D electronics on arbitrary surfaces, which can serve as a complementary technology to photolithography in the planar circuit industry.

## Graphical abstract

A new method called Stamping Lithography for three‐dimensional (3D) circuits manufacturing is proposed, which comprises a resist mask stamping process based on self‐assembly of colloidal particles and a subsequent etching process. It replaces the photoresist with self‐assembled particles and the exposure process with stamping, making it feasible to operate on arbitrary surfaces without the need for sophisticated equipment.

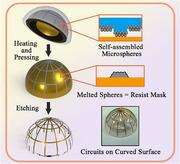

## 1 Introduction

Three‐dimensional (3D) circuits on curved surfaces offer advantages such as a large integration area and excellent spatial adaptability, enabling better coordination and integration with the curved geometries that are widely present in industrial equipment.^[^ ^1^, ^2^, ^3^ ^]^ They have been extensively applied in areas including conformal antennas,^[^ ^4^, ^5^, ^6^ ^]^ intelligent skins,^[^ ^7^, ^8^, ^9^ ^]^ metasurfaces,^[^ ^10^, ^11^ ^]^ and bionic electronics.^[^ ^12^, ^13^ ^]^ While maintaining surface conformity and high precision, the manufacturability, efficiency, and stability of curved‐surface circuits critically influence their practical engineering applications.^[^ ^14^ ^]^

Due to the advantages of high resolution and high throughput, photolithography has been widely used for fabricating micro/nanoscale circuits on planar substrates. However, conventional photolithography encounters significant challenges when it comes to arbitrary curved surfaces.^[^ ^15^, ^16^ ^]^ On the one hand, spin coating, the most common technique for preparing photoresist, often encounters issues on curved surfaces due to the hindrance of radial flow of the photoresist solution by the surface topography, leading to resist accumulation and thickness control difficulties.^[^ ^17^ ^]^ While spray coating can achieve more uniform resist thickness, it often results in significant surface roughness, thus degrading the lithographic resolution.^[^ ^18^ ^]^ On the other hand, extending projection photolithography to non‐planar substrates leads to more pronounced diffraction effects due to the curved surface not being on the same focal plane, further limiting the minimum feature size due to widening of the diffraction rays.^[^ ^19^ ^]^ 3D holographic lithography^[^ ^20^, ^21^, ^22^ ^]^ and curved direct laser writing lithography^[^ ^23^, ^24^, ^25^ ^]^ can improve resist mask resolution by matching the exposure to the curved surface. However, these methods rely on complex optical or motion systems, which limit the manufacturing area and efficiency of curved circuits, making them difficult to scale up for industrial production.^[^ ^26^, ^27^, ^28^ ^]^

Printing manufacturing methods, such as screen printing, inkjet printing, and microcontact printing, are considered more suitable strategies for large‐area curved‐surface manufacturing.^[^ ^29^, ^30^ ^]^ However, the use of ink limits the range of available materials, and factors such as ink viscosity, capillary effects, and the “coffee‐ring effect” caused by solvent evaporation restrict further improvements in linewidth and resolution.^[^ ^31^, ^32^ ^]^ In addition, the poor adhesion of certain ink materials to specific substrates fails to meet the bonding requirements of curved‐surface circuit devices, thereby limiting the industrial applicability of these approaches.^[^ ^33^, ^34^ ^]^

To avoid the disadvantages of the conductive ink, methods involving the transfer of circuit patterns or photoresist patterns onto curved substrates using stamps have attracted widespread attention, owing to the potential for rapid stamp fabrication and reusability, enabling large‐scale production of structured surfaces.^[^ ^35^, ^36^ ^]^ However, they still involved planar stamps to fit curved surfaces, which faced the challenges of excessive stretching when dealing with non‐developable curved surfaces.^[^ ^37^, ^38^ ^]^

Colloidal particle self‐assembly, a simple, efficient, and bottom‐up approach to constructing coatings,^[^ ^39^ ^]^ can adapt to complex curved surfaces. By employing the stamping process, colloidal particles can be transferred to other substrates, forming a large‐area patterned colloidal coating, with resolution down to several particle diameters. This approach has already demonstrated great potential in fields such as materials development, multimodal anti‐counterfeiting, circuit fabrication, and biosensing.^[^ ^40^, ^41^, ^42^, ^43^, ^44^ ^]^ Therefore, integrating colloidal nanoparticle self‐assembly with pattern transfer techniques on complex curved surfaces provides a more efficient strategy for fabricating high‐resolution patterns on arbitrary curved substrates compared to conventional curved‐surface photolithography, while also overcoming the linewidth limitations caused by liquid‐based processes in printing methods such as inkjet printing. The transfer of self‐assembly of particles from the stamp to the substrate, utilizing the adhesion differences between different interfaces, has been proposed in previous literature.^[^ ^41^, ^42^ ^]^ However, there is a lack of research on the transfer mechanism and its exploration in circuit fabrication.

Herein, this paper proposes a new method called Stamping Lithography, which comprises a resist mask stamping process based on self‐assembly of colloidal particles (RSSA) and a subsequent etching process similar to those used in planar circuit technologies. The resist masks made of colloidal particles can serve as traditional photoresists in subsequent circuit manufacturing processes, such as etching. First, colloidal particle coatings were fabricated using a large‐area self‐assembly strategy based on liquid‐air interfacial tension gradients, as previously proposed by our group.^[^ ^45^, ^46^ ^]^ Then, a hot stamping process was utilized to achieve patterned fabrication of the resist mask, and methods to improve transfer efficiency and mask quality were investigated. The self‐assembly process could be carried out directly on a non‐planar substrate,^[^ ^47^ ^]^ and the transfer stamps could be made into shapes that conform to the target surfaces, effectively avoiding defects caused by the deformation of the stamp during transfer. Finally, a touch switch circuit was fabricated on a hemispherical curved surface, demonstrating a successful strategy for preparing circuits on arbitrary surfaces. Compared to the traditional photolithography process, Stamping Lithography utilizes the self‐assembly process instead of spin or spray coating, and the stamping process instead of exposure, which makes it independent of shape and allows it to perform on any surface, including non‐developable ones. Furthermore, the formation of such resist mask layers does not rely on developers, thereby avoiding issues caused by incompatibility between developers and substrates. Benefiting from the use of transfer stamps, the RSSA process can be carried out efficiently in large quantities for various materials, demonstrating potential for mass production similar to traditional photolithography.

## 2 Results and Discussion

### 2.1 Validation of the Stamping Lithography Process on a Planar Surface

**Figure 1:**
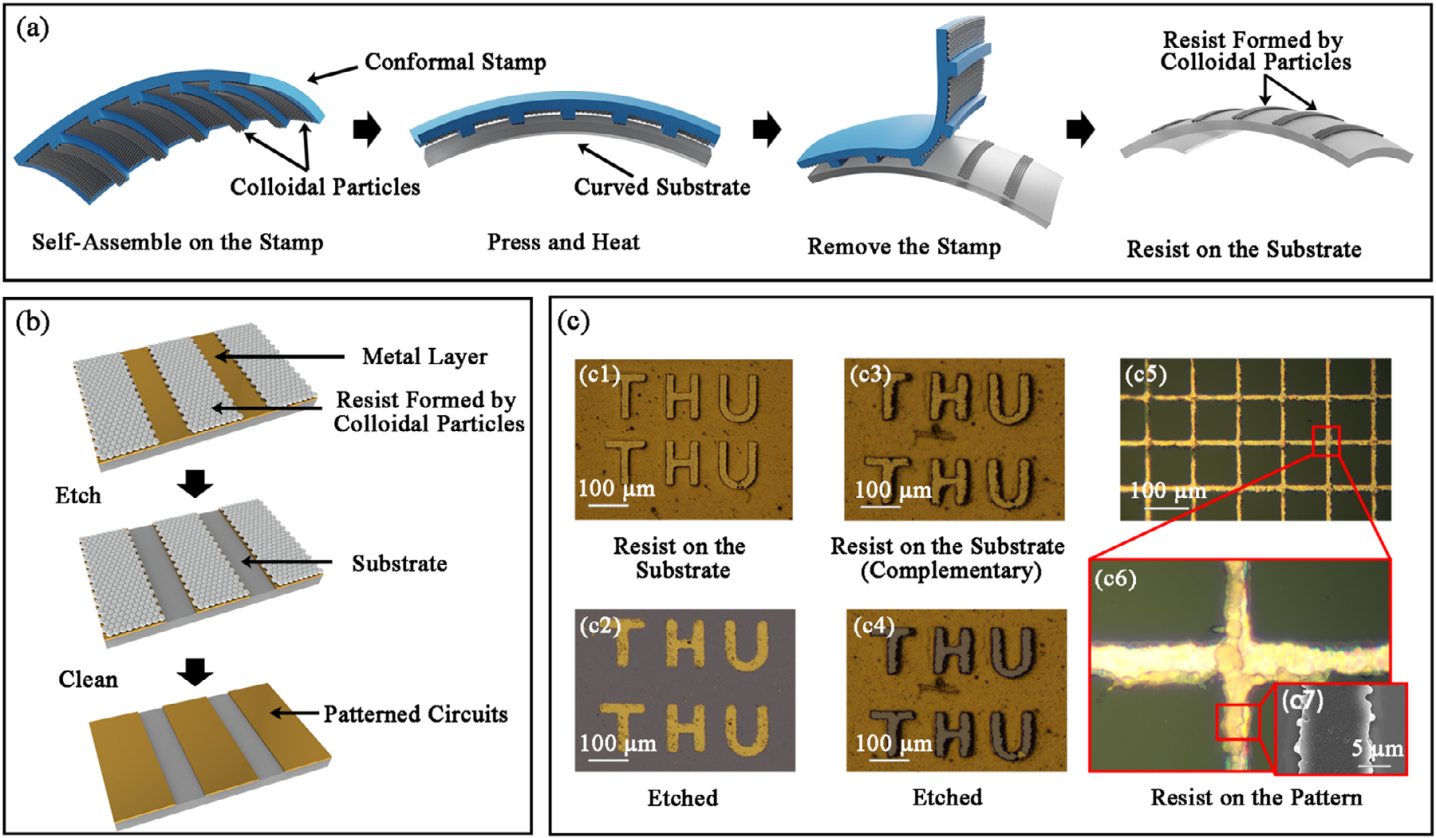
The schematic diagram of Stamping Lithography. a) Schematic illustration of the RSSA process for 3D circuit fabrication. First, colloidal particles were assembled onto a stamp that was conformal to the curved substrate. The stamp had pre‐designed pattern protrusions on the surface. After the pressing and heating process, the particles on the protrusions of the stamp would remain on the surface of the substrate, forming the resist mask with the pre‐designed pattern. b) Schematic diagram of patterned etching based on the resist mask obtained by the RSSA process. c) The experimental results of Stamping Lithography. c1) The resist mask of the microspheres formed the word “THU”. c2) The etched result of (c1), which showed that the resist protected the metal beneath. c3) The resist mask formed a pattern complementary to that in (c1). c4) The etched result of (c3). c5) Preparation of a 10 µm line‐width metal mesh grid was achieved using PS microspheres with a diameter of 1 µm. c6) Magnified views of the resist mask of (c5). c7) Magnified scanning electron microscope (SEM) image of the resist mask of (c6).

The schematic diagram of the Stamping Lithography process flow is shown in **Figure** **1**a. The colloidal particles, polystyrene (PS) microspheres in our experiments, were first assembled on the stamp by any kind of self‐assembly process. The stamp had pre‐designed pattern protrusions on the surface and could be an irregularly curved surface to conform to the target substrate. Either the stamp or the substrate needed to be flexible for subsequent pressing and separation steps. The planar polydimethylsiloxane (PDMS) stamp was achieved by reverse molding a silicon wafer with a SU‐8 photoresist structure, while the curved stamp was obtained by reverse molding a computer numerical control (CNC)‐machined metal mold. The stamp with the particles was then pressed onto the target substrate and kept at a certain pressure and temperature for an adequate period. After separating the stamp and the target substrate, the particles at the protrusions of the stamp would remain on the surface of the substrate to realize the pattern‐based transfer process due to the difference in adhesion of particles between the stamp and the substrate. If the adhesion of the particles to the stamp was stronger, particles could be self‐assembled on the substrate first and then perform the “pick up” action to obtain the patterned resist mask in the same way by heating and pressing. The transferred particles could serve as a resist mask for the following etching process, as shown in Figure 1b. Further etching experiments based on these resist masks demonstrated the effectiveness of the resist masks formed by the microspheres, as shown in Figure 1c. The preparation of a 10 µm line‐width metal mesh grid was achieved using PS microspheres with a diameter of 1 µm. It is believed that with the development of self‐assembly technology, the use of smaller particles will enable the processing of nanoscale feature sizes in the future.

### 2.2 Analysis of the Stamping Process

**Figure 2:**
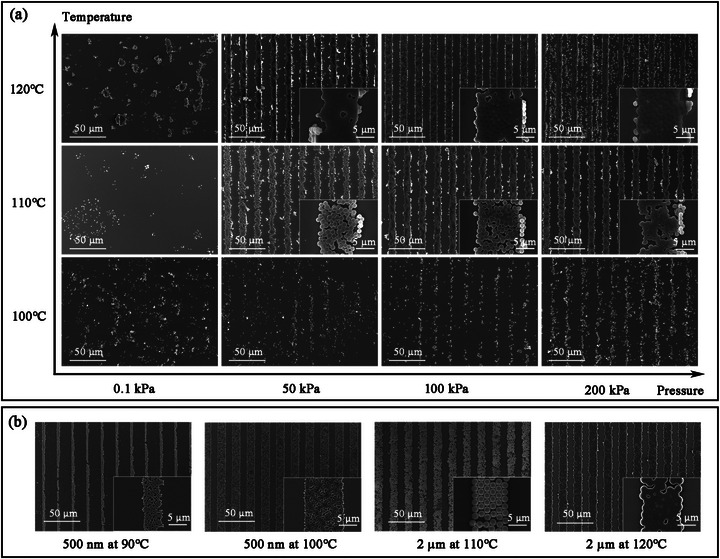
Transferred resist masks by RSSA under different parameters. a) The transfer results of microspheres with a diameter of 1 µm under different temperatures and pressures. The pressure was calculated based on the actual contact area. The 0.1 kPa meant that there was no external pressure except for the self‐weight of the stamp. b) Transfer results of microspheres with diameters of 500 nm and 2 µm at different temperatures. The pressure was 100 kPa.

To obtain the optimal transfer parameters, we investigated the influence of different factors on the morphology of the transfer pattern, as shown in **Figure** **2**a. PS microspheres with a diameter of 1 µm were used as the self‐assembly material, and the micro‐pattern on the stamp was a line array with a width of 10 µm. The selection of such a size allowed for observation of the overall morphology of the transferred lines while also focusing on the specific changes of every single microsphere. The phenomenon of transfer could only occur when pressure and heating were both applied. It could be observed from the subgraph that the particles after stamping at 110 °C still maintained their spherical shape, while the particles after stamping at 120 °C showed obvious signs of melting and re‐bonding. The pressure took into account the real contact area, i.e., the area of the patterned protrusions contacting the substrate rather than the total area of the stamp. For example, the stamp had a total area of 4 cm^2^, but 50% of the area was the protrusions of the line array pattern. Therefore, the real contact area of the stamp was 2 cm^2^, and the corresponding pressure was 100 kPa instead of 50 kPa when the applied external force was 20 N.

To further demonstrate the universality of RSSA for resist mask preparation, PS microspheres of different sizes were chosen for the same experiment, and the results are shown in Figure 2b. When 500 nm microspheres were stamped at 90 °C, the microspheres transferred to the target surface, but the pattern was not complete, which was similar to the situation of 1 µm microspheres at 100 °C. This indicated that smaller‐sized microspheres require a lower transfer temperature, and the transfer temperature required for 2 µm microspheres was close to that for 1 µm microspheres. This was consistent with common sense that the smaller the size of the polymer material, the more likely it is to melt at low temperatures. It could be inferred that both larger and smaller microspheres could be used in the Stamping Lithography process, and the use of smaller microspheres was expected to perform at low temperatures, thereby enabling circuit manufacturing on organic flexible substrates. Considering that the self‐assembly process of microspheres with different sizes required specific optimization, this paper mainly took 1 µm microspheres as examples.

**Figure 3:**
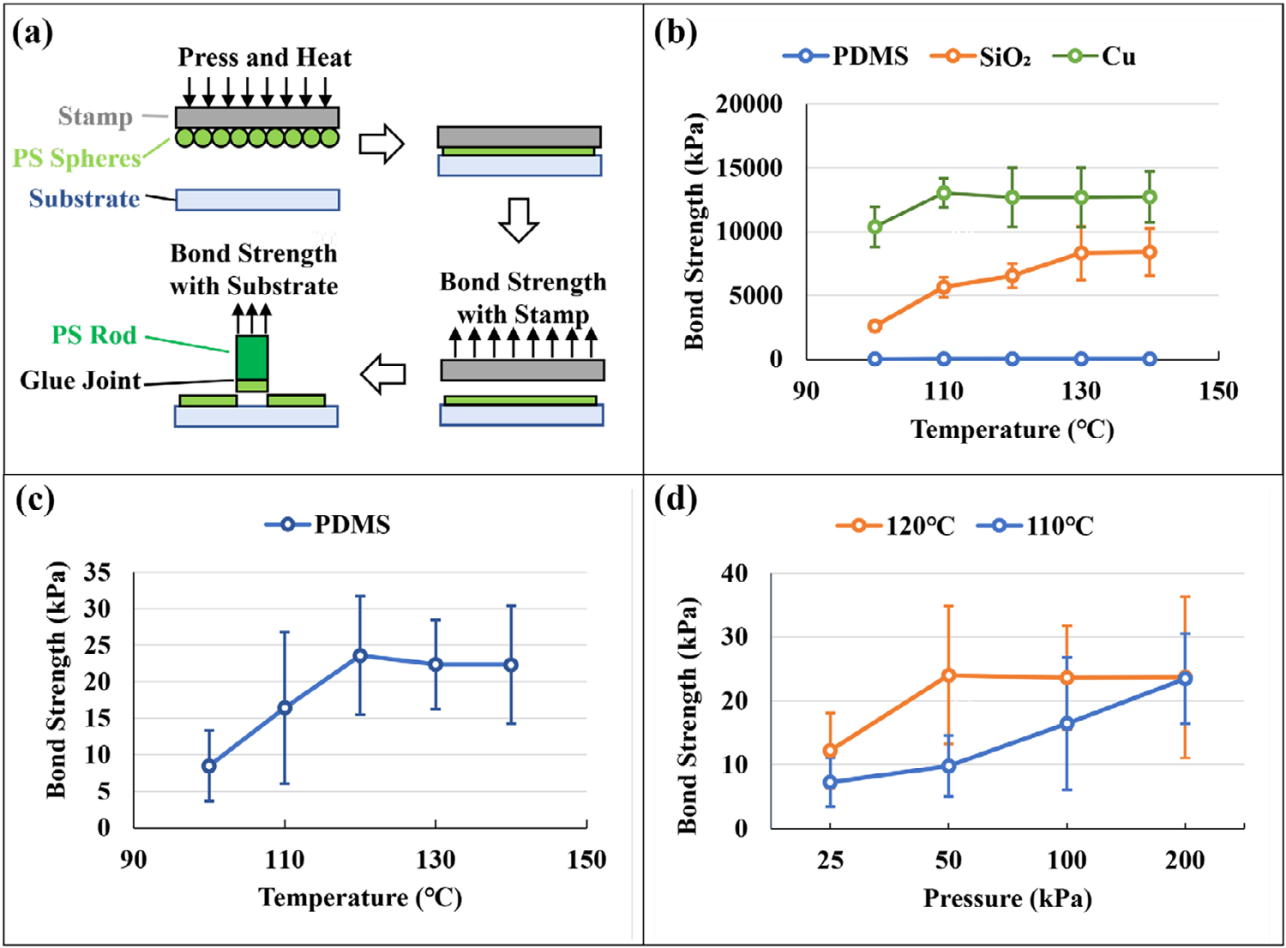
Bond strength of the PS spheres between different materials. The measurements were taken after cooling down to room temperature. a) The schematic diagram of the bond strength measurement method. The bonding force between the PS microspheres and the stamp was measured through the separation process after hot pressing. The bonding force between the PS spheres and substrate was measured by a traditional paint adhesion pull‐off test after stamping. b) The bond strength of PS between different materials under different temperatures. c) The bond strength with the PDMS stamp under different temperatures. d) The bond strength with the PDMS stamp under different pressures and temperatures.

Furthermore, we investigated the influence of temperature and pressure parameters on the physical mechanism of the stamping process through a tensile test measuring the bond force of “particles versus substrate” and “particles versus stamp”. As shown in **Figure** **3**a, the pressing and heating process was conducted as usual, but the removal of the stamp was done on the tensile test machine. The maximum force during the separation of the stamp was denoted as the bond strength with the stamp. The bond strength between the PS microspheres and the substrate was measured by the conventional pull‐off test for measuring paint adhesion. A PS rod was glued on the layer of PS microspheres transferred onto the substrate, and the force needed to pull off the rod from the substrate was denoted as the bonding strength with the substrate. Figures S1 and S2 (Supporting Information) showed that the layer of PS microspheres was transferred completely onto the substrate and pulled off together with the PS rod. For more experimental details, please refer to Figures S3–S5 (Supporting Information). The above measurements were taken after cooling down to room temperature. To investigate the effect of temperature on stamping, PDMS stamps, quartz glass substrates, and copper substrates were tested, as shown in Figure 3b. The bond strength with the substrate outweighed the bond strength with the stamp by ≈2 orders of magnitude, which proved the reliability of selecting PDMS material as the transfer stamp. The bond strength with the PDMS stamp is shown separately in Figure 3c. All curves of the bond strength showed a clear trend of increasing first and then stabilizing after reaching a certain temperature, indicating that there was a temperature transition point where the melted particles came into complete contact with the surface. This transition point was related to the material, as shown in Figure 3b,c, caused by the wettability of different material surfaces. Further experiments had shown that this turning point was not only related to temperature, but also to transfer pressure, as shown in Figure 3d. The bond strength tests between PS microspheres and PDMS stamps under different external pressure conditions were conducted at 110 and 120 °C, respectively. Similar to temperature, the pressure curve also had a transition point. Before reaching this transition point, the bonding strength increased with the pressure, and after reaching the transition point, the bonding strength tended to stabilize. Comparing two curves at different temperatures, it could be seen that lowering the temperature would increase the pressure transition point. All of these results were in line with common sense, because polymer particles after heating were viscoelastic materials rather than conventional liquids, and their wetting state with other materials was not only determined by the surface tension of the material, but also affected by external pressure. The higher the temperature, the better their fluidity, so less force was needed to achieve sufficient contact with other material surfaces at higher temperatures. The bond strength finally reached a stable value under sufficient pressure at different temperatures, as shown in the two curves in Figure 3d, which further confirmed this conclusion. The bond strength was related to the real contact area and surface energy. Since the measurements were conducted at room temperature, the stamping temperature and external pressure did not affect the surface energy directly, but rather altered the magnitude of the binding force by affecting the real contact area. Therefore, when the softening deformation of the particles compensated for the roughness difference between the two surfaces and achieved complete contact, the binding force reached the same stable value.

Considering that most substrate materials had limited heat resistance, it was reasonable to use lower temperatures as much as possible. Therefore, unless otherwise specified in the subsequent text, 120 °C was used for stamping. Although too much pressure would not affect the bond strength, it could cause a series of engineering problems, such as severe deformation of the stamps and excessive reduction in thickness of the resist mask, which would reduce the quality. Therefore, unless otherwise specified in the subsequent text, 50 kPa of pressure was used.

It could be concluded that the physical properties of particles at the micrometer scale were consistent with those of macroscopic materials, while the viscous flow temperature of PS microspheres dropped significantly to ≈100 °C. Macroscopic PS materials typically had a glass transition temperature of ≈100 °C and a viscous flow temperature of ≈200 °C. When heated above the glass transition temperature, it would enter a highly elastic state, similar to rubber with good elasticity, but would return to its original shape after removing external force. After being heated above the viscous flow temperature, it exhibited both elasticity and fluidity and underwent irreversible deformation when subjected to external forces. Only after reaching this state could the transfer be possible.

When the experiment was conducted at room temperature, no matter how much the pressure was increased, the transfer results could not be improved. The stamp and substrate surfaces were not completely flat, and the size of the particles varied slightly. Therefore, when self‐assembling on the stamp, the contact between the particles and the stamp was better, with a larger actual contact area. When pressed at room temperature, the particles remained in a hard glassy state, with minimal deformation during the pressing, making it difficult to fully contact the target substrate. Therefore, stamping cannot be performed at room temperature. The key to achieving complete transfer was to make the particles enter the viscous flow state and undergo softening deformation, so as to compensate for the roughness and fully contact the two surfaces.

### 2.3 Strategies for Improving the Quality of the Transferred Resist Mask

The ideal patterned resist mask should have no defects inside. However, affected by thermodynamic fluctuations, the self‐assembly process was difficult to achieve a large area of flawless, close‐packed hexagonal structure, resulting in defects in the resist mask after transfer. Although many researchers were devoted to proposing new methods to improve the quality of self‐assembly colloidal particles, more robust methods should be proposed to improve the quality of the transferred resist mask in order to achieve good practical applications under common conditions.

**Figure 4:**
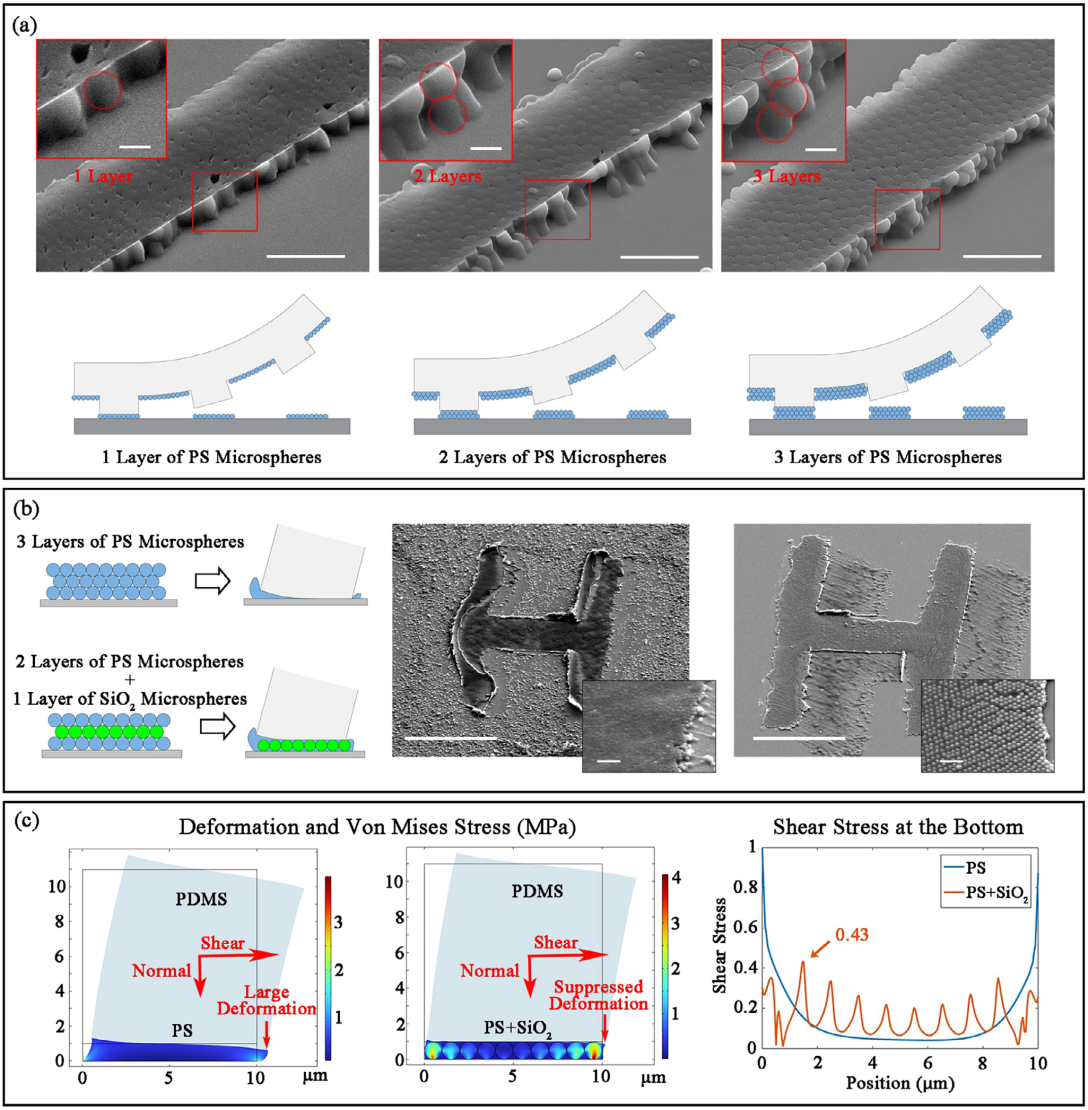
Optimization of resist mask quality. a) Minimizing the defection due to thermodynamic defects during self‐assembly by increasing the number of microsphere layers. With the increase in the number of microsphere layers, the defects in the previous layer of microsphere resist mask would be filled by the next layer. Scale bar, 5 µm. The insets demonstrated that the number of self‐assembled layers could be clearly observed on the edges. Scale bar, 1 µm. b) By adding high‐temperature‐resistant particles, such as silica, the mechanical strength of the mask could be effectively improved. The SEM images on the left and right correspond to the resist mask formed after a non‐ideal pressing process of “3 layers of PS spheres” and “2 layers of PS spheres + 1 layer of SiO_2_ microspheres”, respectively. Scale bar, 100 µm. The insets show a partial magnification of the resist mask, respectively. Scale bar, 5 µm. c) The finite element simulation of the shear resistance of self‐assembled layers with/without SiO_2_ microspheres added. The self‐assembled layer with SiO_2_ added had significantly suppressed deformation under the same shear force. The shear stress curve at the bottom indicated that a pure PS layer would generate a larger shear force, which was very likely to cause slippage during transfer. The SiO_2_ microspheres evenly dispersed the shear stress, reducing the maximum value of the shear stress to less than half, significantly reducing the possibility of slippage.

The resist mask quality could be greatly improved by multi‐layer self‐assembly, and the same protective effect as traditional photoresist could be achieved. Since most self‐assembly methods could achieve multi‐layer self‐assembly, a single transfer operation was sufficient to transfer multi‐layer particles, effectively avoiding precision reduction caused by multiple transfers. As shown in **Figure** **4**a, from the morphology of the edges of the transferred resist mask, it was obvious that the multi‐layer particles could be completely transferred to the target substrate without remaining on the stamp, which indicated that the binding force among the particles was greater than the adhesion force between the substrate and stamp. However, an insufficient transfer time resulted in insufficient connection among the particles, causing them to disconnect from the middle when the stamp was removed, leaving the particles on the stamp, as shown in Figure S6 (Supporting Information).

Considering the actual situation of the pressing process was not ideal, such as due to the high local pressure caused by an uneven pattern of the stamp, or the lateral movement caused by thermal expansion, or non‐vertical pressure caused by pressing on a curvy surface, the shape of the transferred resist pattern would undergo excessive deformation, as shown in Figures S7 and S8 (Supporting Information). By adding high‐temperature‐resistant particles, such as silica, the strength of the resist mask could be effectively improved, which was conducive to maintaining the basic shape of the resist mask with a specific thickness after non‐ideal pressing, as shown in Figure 4b. The SEM images on the left and right correspond to the resist mask formed after a non‐ideal pressing process of “3 layers of PS spheres” and “2 layers of PS spheres + 1 layer of SiO_2_ microspheres”, respectively. The resist mask completely composed of PS, was seriously deformed during the pressing process; the original straight line had been bent, and the edge of the mask had warped. If the etching process were carried out later, the flowing etching liquid would wash away the entire resist mask, greatly damaging the quality of the pattern. Moreover, due to the uneven pressure or temperature distribution under certain conditions, the resist mask thickness in the high‐pressure or high‐temperature area was thinner if only the PS microspheres were used. However, the resist mask with SiO_2_ added still maintained the basic morphology with a certain thickness after excessive pressing, and the warping phenomenon at the edge was suppressed. Although there were traces of PS left during the lateral movement, these traces would not form a dense resist mask, and the etching process could still proceed normally, as shown in Figure S9 (Supporting Information). The simulation of the shear resistance of self‐assembled layers with/without SiO_2_ microspheres added is presented in Figure 4c. The simulation considered the case of the PDMS stamp and the underlying self‐assembled layer. For simplicity, it was assumed that the PS microspheres had melted and formed a homogeneous mixture with the SiO_2_ microspheres. Compared with pure PS, the self‐assembled layer with SiO_2_ microspheres added had significantly suppressed deformation under the same shear force. The shear stress distribution at the bottom was shown in the curve, indicating that a simple PS layer would generate a relatively large shear force at the edge, which was very likely to cause slippage during transfer. Although SiO_2_ microspheres had created stress concentration points, they evenly dispersed the shear stress in the whole plane, reducing the maximum value of the shear stress to less than half, significantly reducing the possibility of slippage.

Therefore, adding SiO_2_ in the self‐assembly process could greatly improve the quality of the resist mask. Considering that the heating and pressing were difficult to be uniform in the actual process, especially in the case of a non‐planar surface, it might occur that the local temperature or pressure was too high, and the liquidity of the particles might be strengthened. Therefore, the above analysis under non‐ideal pressing conditions was of practical guiding significance.

**Figure 5:**
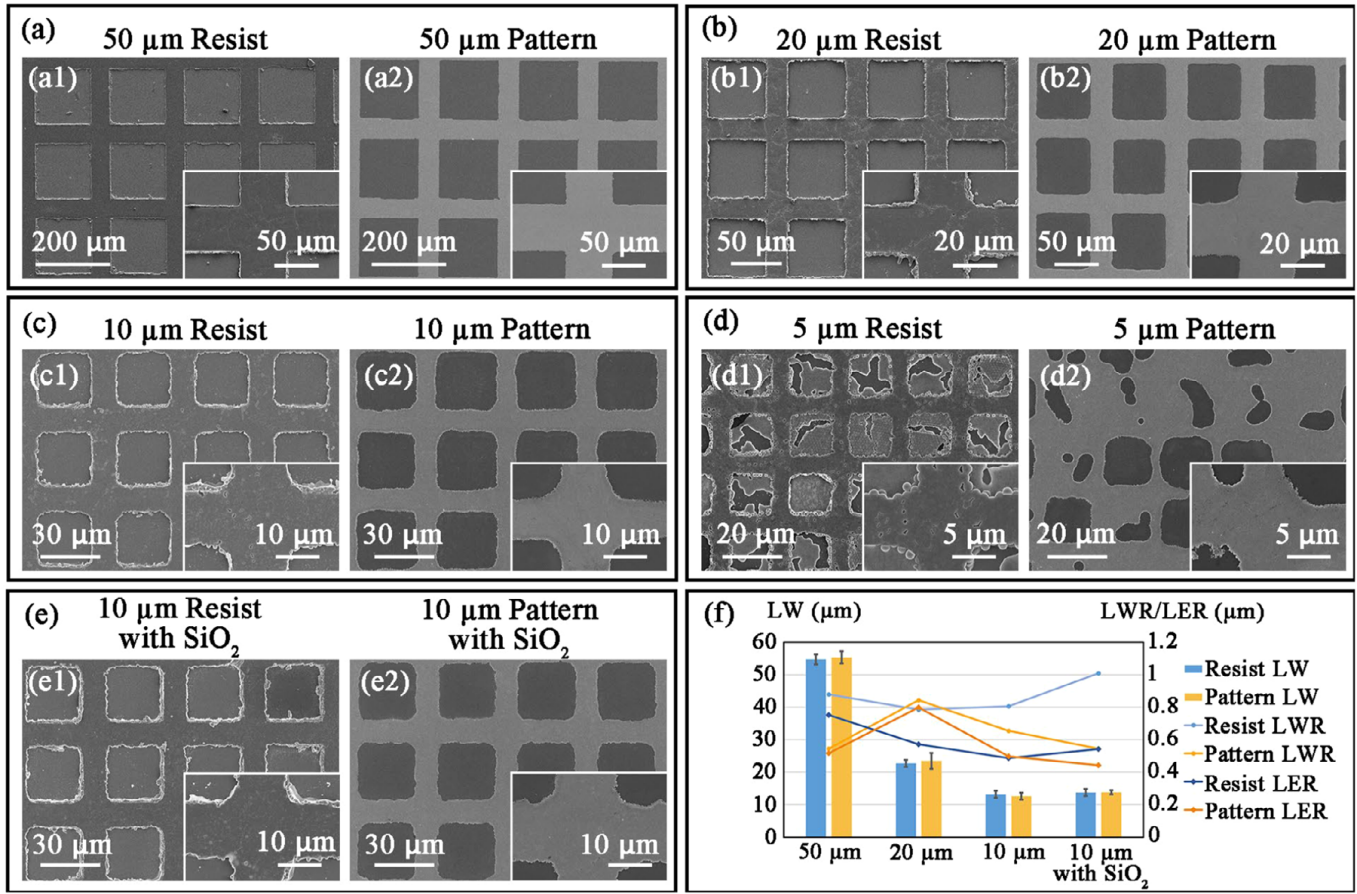
Quality analysis of line patterns obtained by Stamping Lithography. a–d) The resist mask and final etched patterns were obtained by Stamping Lithography on copper‐plated silicon wafers using three layers of 1 µm PS microsphere self‐assembled layers, with line widths of 50, 20, 10, and 5 µm, respectively. Among them, the 5 µm grid was basically incomplete, while the line quality of the 10 µm one was good. e) The resist mask and the etched pattern were achieved with 2 layers of PS spheres and 1 layer of SiO_2_ microspheres in the middle. All microspheres had a diameter of 1 µm. The pattern had a line width of 10 µm. f) Quantitative analysis of pattern quality by Stamping Lithography. LW, line width. LWR, line width roughness. LER, line edge roughness. The quality of the pattern had no obvious relationship with the pattern itself. Both the LWR and LER were approximately the diameter of the microspheres used.

After optimizing the quality of the resist mask, the accuracy of the pattern by Stamping Lithography was tested, as shown in **Figure** **5**. Tests were conducted on copper‐plated silicon wafers using grid structures of different line widths with 3 layers of 1 µm microspheres as the standard. The results of resist masks and final etched patterns with line widths of 50 µm, 20 µm, 10 µm, and 5 µm are shown in Figure 5a–d, respectively. It could be seen that there were no etching marks on the copper surface, indicating that the microsphere mask had achieved a good protective effect. When stamping a 5 µm pattern, due to the use of three layers of PS self‐assembled microspheres with a diameter of 1 µm, many microspheres filled the gaps between the lines. These microspheres extended from both sides of the lines and overlapped, resulting in over‐transfer. Since smaller microspheres could also be transferred, as mentioned before, the pattern could possibly reach feature size on the nanoscale by using nanospheres. From our experimental results, it could be inferred that the Stamping Lithography could easily achieve the processing of a feature size of 10 times the microsphere used, with good quality. Figure 5e shows the resist mask and the etched pattern obtained by using the self‐assembled layer with SiO_2_ added. The results proved that the addition of SiO_2_ did not compromise the final pattern quality. Figure 5f shows the quantitative analysis of the results. The line width (LW) of the pattern did not change significantly before and after etching. However, they were all slightly higher than the design value. This was because the overall width increases after multi‐layer self‐assembly, which could be solved by adjusting the size during the design. The line width roughness (LWR) and line edge roughness (LER) before and after etching had no obvious correlation with the size of the pattern, and their values were slightly smaller than the diameter of the microsphere. All the above results demonstrated the excellent quality of Stamping Lithography in circuit processing, and indicated that higher‐precision processing could be achieved by further optimizing the self‐assembly process and using smaller‐sized microspheres.

### 2.4 Stamping on Curved Surfaces Based on Conformal Stamps

Since the spherical surface is non‐developable, traditional transfer printing processes using planar stamps will encounter the problem of being overstretched and unable to fully contact the non‐developable surface through deformation. Nevertheless, the RSSA process can solve this problem using a curved stamp that is conformal to the target substrate, which can accurately transfer the designed pattern to the target substrate without any deformation.

**Figure 6:**
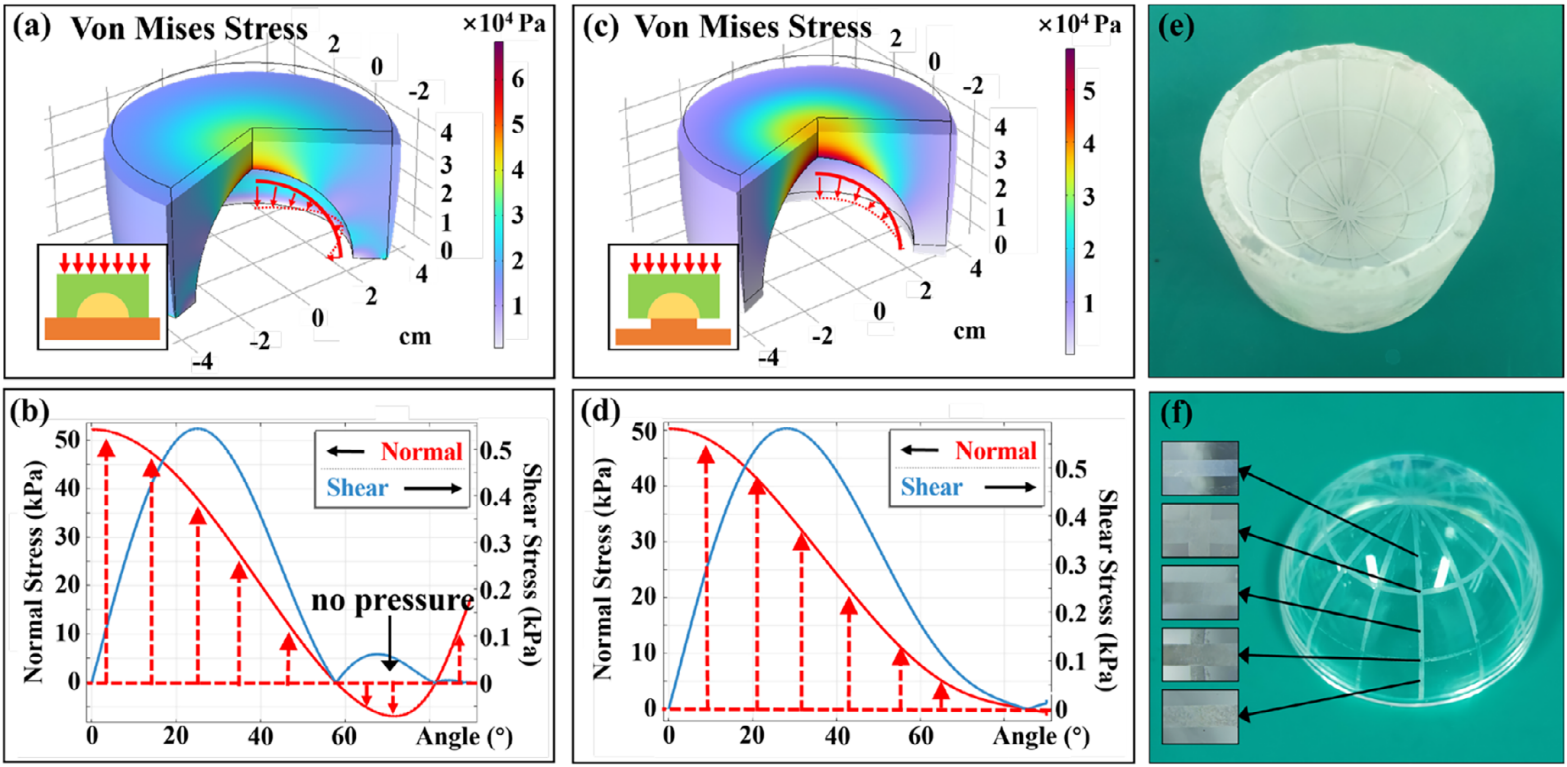
Mechanical Simulation analysis and experimental results of RSSA on the hemisphere surface. a) Mechanical simulation when both upper and lower surfaces were in direct contact with the pressure plate. The color represented von Mises stress. The red arc and arrows in the image were schematic diagrams of the force on the substrate surface. The inset image showed a schematic diagram of the stamping process. The yellow part in the middle was the curved substrate, the green part above was the flexible curved stamp, and the orange part below was the support. b) The normal stress (red curve) and the shear stress (blue curve) on the substrate surface of the red arc in (a). c) Mechanical simulation when the lower surface of the stamp was suspended, as shown in the inset. Other legends were consistent with (a). The green stamp did not contact the orange support. d) The normal stress (red curve) and the shear stress (blue curve) on the substrate surface of the red arc in (c). The insets were actual images of the transfer results at the corresponding positions. Scale bar, 1 mm. e) The actual image of the curved stamp with 3 layers of self‐assembled particles. g) The actual image of the transferred resist mask on the hemisphere substrate.

To demonstrate the feasibility of the RSSA on non‐developable surfaces, we tested its ability on glass hemispheres as an example. According to the previous research on planar stamping, the magnitude of pressure was not the decisive factor in RSSA. Reducing the pressure at higher temperatures could also achieve the same transfer quality. Therefore, even if the pressure distribution was not uniform, as long as a conformal stamp consistent with the shape of the curved substrate was used, a transferred pattern with good quality on the curved surface could be achieved. We prepared PDMS stamps with a flat top and a hemispherical depression at the bottom, and used a common hot press to conduct the stamping process. The molds used for PDMS preparation were made of aluminum alloy by CNC machining. The mechanical simulation during the stamping process is shown in **Figure** **6**a. However, when both upper and lower surfaces were in direct contact with the pressure plate of the hot press, as depicted in the inset image, there would be a region with no pressure, as depicted by the red arc and arrows. The normal pressure on the substrate dropped below zero within the range of 60° to 80°, as shown in Figure 6b, which meant that transfer could not be achieved in this area. This problem could be effectively solved by changing the support method at the bottom, as shown in Figure 6c,d. Only the bottom of the substrate had support, while the bottom of the stamp was in a suspended state, which allowed the stamp to deform downwards and could improve the contact condition of the edges. Although the pressure still decreased near the edge, it could expand the range of positive pressure, thereby increasing the transfer area. The simulation results showed that the shear stress in the stamping process was much smaller than the normal stress. Considering that the shear resistance of the mask layer had been significantly improved after adding SiO_2_, the problems caused by the shear force in this process should be relatively small. The multi‐layer self‐assembled curved stamp is shown in Figure 6e. The final transfer result on the hemisphere is shown in Figure 6f, and the detailed images corresponding to different positions were illustrated as the insets in Figure 6d. It could be seen that the transfer quality did not decrease significantly with the decrease in pressure, which made it possible to prepare circuits on a non‐developable curved surface.

### 2.5 Fabrication of Circuits on Non‐Developable Surfaces

**Figure 7:**
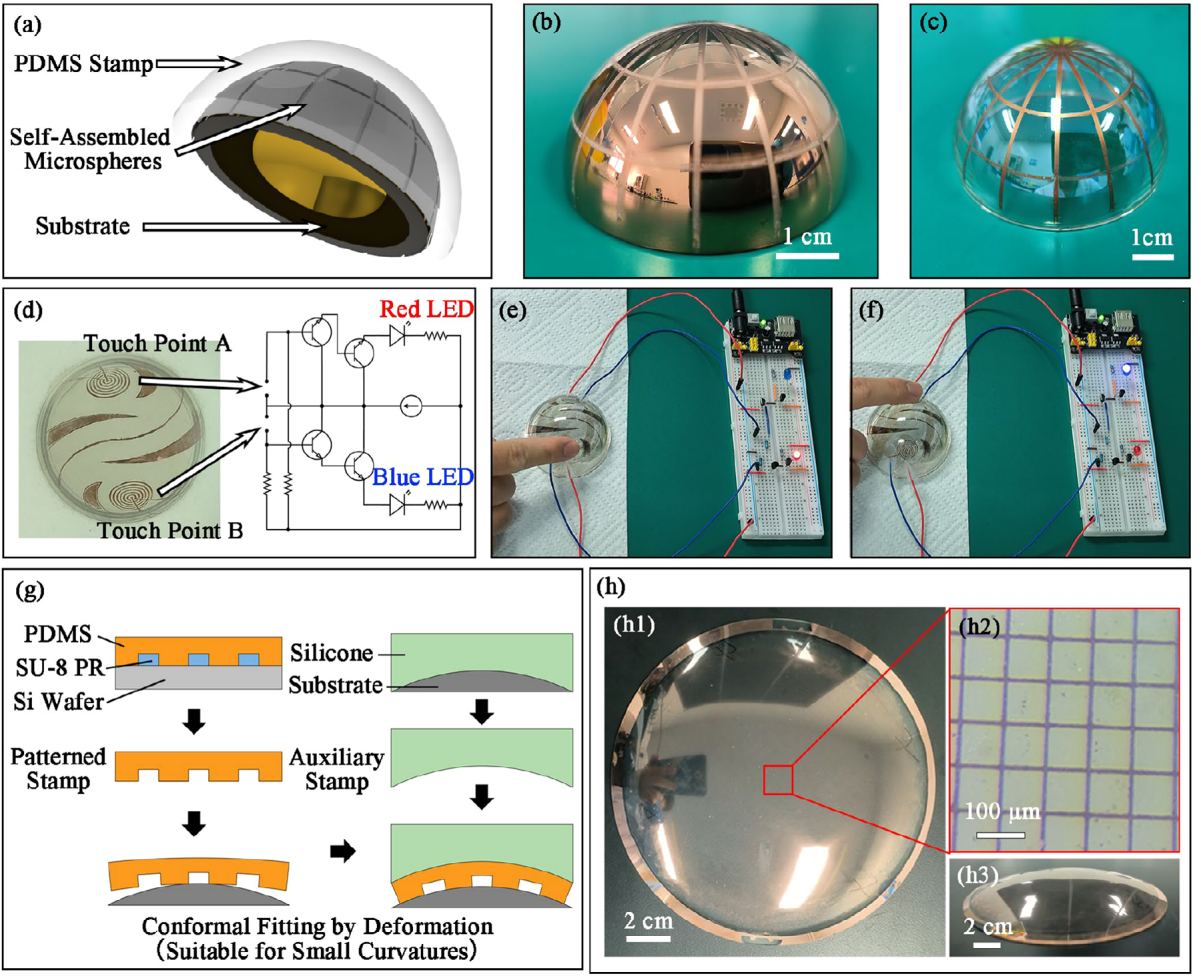
Validation model of functional circuits manufactured on non‐developable surfaces by the Stamping Lithography process. a) Schematic diagram of the RSSA process on a curved surface. b) The resist mask on the copper‐plated glass hemisphere obtained by the RSSA process. c) The final pattern of copper line on the curvy surface after Stamping Lithography. d) Image of a touch circuit prepared on a curved surface and the schematic diagram of peripheral electronic components. e,f) When the two touch points on the 3D circuit were touched by a finger, the corresponding red or blue LED lights would light up. g) A method for high‐precision circuit fabrication applicable to small curvature substrates. High‐precision planar stamps were obtained by utilizing traditional planar lithography and PDMS casting. Prepare conformal auxiliary stamps by silicone. Relying on the flexibility of PDMS itself, the stamp fitted the curved surface during the stamping process. PR, photoresist. h) A copper grid sample with a line width of 10 µm on a large‐area non‐developable curved surface. h1) Top view of the sample. h2) Microscopic image of the grid. h3) Side view of the sample.

To further validate the potential of Stamping Lithography for 3D electronics, we fabricated simple circuits on glass hemispheres as an example. On the hemispherical surface of quartz glass, a 100 nm thick metallic copper layer was prepared on the surface by magnetron sputtering. As shown in **Figure** **7**a, the height of the protrusion on the stamp was much greater than the thickness of the self‐assembled microsphere layer, causing only the protrusion to come into contact with the substrate when pressing the stamp with the target. The resist mask prepared on the hemispherical surface by the RSSA process is shown in Figure 7b. The resist mask was then used for traditional wet etching, and finally, the patterned copper lines on the curved surface were obtained, as shown in Figure 7c. To further illustrate the feasibility of the 3D circuit, we designed a touch switch circuit on the curved surface and connected it with peripheral electronic components such as LED lights, as shown in Figure 6d. There were two groups of concentric circles on the curved surface as touch points. When a finger touched, the circuit at the concentric circles would be connected to light up the corresponding color LED, as shown in Figure 7e,f. The physical demonstration video could be seen in Video S1. However, the Stamping Lithography process was limited by the processing accuracy of curved stamps, which relies on other micro/nano manufacturing technologies. We proposed a simple scheme as an alternative, which, although only suitable for small‐curvature substrates, could achieve the manufacturing of high‐precision patterns, as shown in Figure 7g. The high‐precision planar stamp was obtained by utilizing traditional planar lithography and PDMS casting. A conformal auxiliary stamp was made of silicone. Relying on the flexibility of PDMS itself, the patterned stamp fitted the curved surface during the stamping process when pressing between the substrate and the auxiliary stamp. A copper grid sample with a line width of 10 µm on a large‐area non‐developable curved surface was achieved through this method, as shown in Figure 7h. These results strongly prove the possibility of preparing 3D circuits on curved surfaces by Stamping Lithography. Compared with other surface lithography technologies, such as holographic photolithography or nanoimprint lithography, Stamping Lithography has the advantages of a simple process flow and good adaptability to surfaces with any curvature.

## 3 Conclusion

In conclusion, aiming to address the difficulty of the traditional lithography process in preparing patterned resist masks on curved surfaces, we propose a novel method for preparing patterned circuits on arbitrary shapes. This method utilizes melted self‐assembled particles as a resist mask and replaces the exposure steps in photolithography with a stamping process. The self‐assembly process is easy to perform and can produce a uniform film on an arbitrary curved surface, thereby avoiding the problem of spinning coating on non‐planar surfaces. The transfer process utilizes a stamp that conforms to the target substrate, minimizing additional tensile deformation during the pressing process. This solution addresses the issue that traditional transfer printing with planar stamps is not suitable for surfaces with large curvature. The circuit is manufactured in situ, avoiding damage to the circuit itself during the stamping process. The protective capacity of the patterned resist mask formed by self‐assembled colloidal particles was verified, making it applicable to the etching process as a traditional photoresist. Further analysis of the formation conditions of the resist mask reveals that resist mask transfer only occurs when the temperature exceeds the viscous flow temperature and the particles undergo sufficient deformation to achieve full contact with the substrate. In addition to controlling transfer parameters, other methods to improve resist mask quality have also been proposed and analyzed. For example, multilayer particles can be transferred by one step and form a multilayer resist mask to compensate for the defects caused by thermodynamic fluctuations in the process of self‐assembly. It should be pointed out that when multilayer particles are being transferred, they need to maintain enough time to make all particles melt together and form a whole layer. In addition, the composite self‐assembly structure can be formed by introducing particles of other materials to improve the mechanical strength of the resist mask, which is conducive to maintaining the morphology and thickness. Finally, a simple circuit was prepared on the surface of hemispherical glass, which verified its basic function and further proved the feasibility of Stamping Lithography for preparing 3D curved circuits. A feasible method for patterned resist mask preparation on an arbitrary surface is proposed, which is compatible with the conventional planar circuit technologies. Compared with in‐situ additive manufacturing technologies such as inkjet printing for curved surfaces, Stamping Lithography has the advantages of high efficiency and larger work areas, which has potential for large‐scale production. Compared with other soft lithography processes, such as nanoimprint lithography, Stamping Lithography uses solid particles instead of liquids, avoiding the defects caused by liquid flow on curved surfaces. Aiming to obtain higher‐quality resists, how to achieve uniform vertical pressure distribution and temperature control on curved surfaces is a topic worthy of further study. It is believed that this technology will gain more attention in the production of 3D circuits.

## 4 Experimental Section

### Materials

The sodium lauryl sulfate (SDS), acrylamide, N, N″‐methylene bisacrylamide (MBA), ammonium persulfate, N, N, N″, N′‐tetramethylethylenediamine, and ammonia solution were purchased from Aladdin Industrial Co., Ltd. (China). The polystyrene nanoparticle colloidal dispersion (2.5% w/v) was purchased from Tianjin Baseline Chromtech Research Center (Tianjin, China). The silicon wafers were purchased from Suzhou Ruicai Semiconductor Co., Ltd. The quartz glass plates and hemispheres were purchased from Donghai Yibo Quartz Products Co., Ltd. The PDMS kits (Sylgard 184) were purchased from Dow Corning Co., Ltd.

### The Preparation of the SDS Hydrogel

The acrylamide (0.03 mol), MBA (0.19 mmol), the redox pair of ammonium persulfate (0.07 mmol), N, N, N', N'‐tetramethyl ethylenediamine (10 µL), deionized water (20 mL), and a specific weight of SDS were mixed to form hydrogel precursors containing the specific content of SDS. The solution was mechanically stirred for 1 h and then transferred to molds for 10 h at 30 °C to fully cross‐link. Then, it was transferred to an oven at 70 °C for 24 h to fully dry.

### The Preparation of the Stamps

The curing agent and main agent in the PDMS kit were mixed at a ratio of 1:10 and poured into the mold. Cured at 80 °C for 2 h. For planar substrates, the mold was made from a silicon wafer patterned by photolithography. For curvy substrates, the conformal PDMS stamps were prepared by an aluminum alloy mold, which was obtained by CNC machining.

### Tension Gradient‐Induced Self‐Assembly

The microsphere monolayer was prepared using the tension gradient‐induced self‐assembly strategy. The detailed steps can be found in our previous research.^[^ ^45^, ^46^ ^]^ Here, we provide a summary. First, SDS hydrogels that allow controlled release of SDS were prepared. Hydrophobically modified polystyrene microsphere dispersions were then added dropwise into a container filled with deionized water. The prepared SDS hydrogel was placed at one end of the container, causing the slow release of SDS into the water. This created a surface tension gradient, which led to Marangoni flow. As a result, the particles gathered at the opposite end of the container and formed a closely packed single‐layer film at the liquid‐gas interface. The substrates were then immersed in the liquid below the monolayer of particles and gently lifted out, thus completing the transfer of the particles from the liquid‐air interface to the substrate material. To obtain a multi‐layer self‐assembled structure, wait for the substrate to completely dry and repeat the self‐assembly process.

### The Stamping of the Particles

The stamp with particles self‐assembly was placed on the target substrate and put into a vacuum hot press together. To achieve more even pressure, a soft silicone pad could be stacked on top of it. The stamping parameters, such as temperature, time, and pressure, were controlled by the hot press, and the vacuum was maintained throughout the transfer process. After the hot pressing, the air pressure was restored, and the stamp and the substrate were taken out to cool at room temperature. The stamp was removed after completing the cooling process.

### The Etch Process

After the stamping, the particles formed a resist mask with the same performance as photoresist in photolithography, which provided efficient protection for the layers beneath. The operation of the etching process was similar to that of the traditional process. For the etching process, the etching solution was sprayed until the pattern was fully visible, then the substrate was cleaned with deionized water and dried with nitrogen gas. The resist mask formed by polystyrene microspheres was removed by gently wiping with toluene or other organic solvents.

### Measurement of Bond Strength with the Stamp

In order to facilitate subsequent measurements on the tensile testing machine, the PDMS stamp was bonded to the glass slide before stamping. The stamp and the glass slide were put into the Plasma Cleaning Machine for 15 s at 40 W. Then they were pressed together for 10 s to fully bond. A set of clamps for measuring bonding strength on the tensile testing machine was designed. The stamping process was performed as described above, but the stamp was placed on a tensile testing machine before the final stamp separation step. The stamp was lifted by the tensile testing machine, and the tensile curve was recorded until the stamp was separated from the substrate. The maximum tensile strength was denoted as the bonding strength with the stamp. More detailed information could be found in the Supporting Information.

### Measurement of Bond Strength with Substrate

After the stamping, the side with transferred PS microspheres on the substrate stamp was glued with a PS rod by the PS‐specific glue. They were set on the tensile machine after 24 h so that the glue was completely dry. Then the rod was lifted by the tensile testing machine, and the tensile curve was recorded until the rod was separated from the substrate. The maximum tensile strength was denoted as the bonding strength with the substrate. More detailed information could be found in the Supporting Information.

### Design of the Switch Circuit on the Hemisphere Substrate

The circuit of the touch switch adopts the simplest resistive switch. The contact point was originally two unconnected metal strips. When a finger touches them, the circuit conducts and generates an electric current. This current activated the transistor, thereby lighting up the LED. For detailed circuit design diagrams, please refer to Figure S10 (Supporting Information).

## Conflict of Interest

The authors declare no conflict of interest.

## Supporting information



Supporting Information

Supplemental Video 1

## Data Availability

The data that support the findings of this study are available in the supplementary material of this article.
