# Supplementary material for: Stamping Lithography on Arbitrary Surfaces based on Self‐Assembly of Colloidal Particles
Source: Adv Sci (Weinh). 2025 Nov 29;13(9):e18380. doi: 10.1002/advs.202518380 (PMC12904012; doi:10.1002/advs.202518380)
Supplement: Supplementary file 1 — Supporting Information [file ADVS-13-e18380-s001.docx]

Supporting Information

Stamping Lithography on Arbitrary Surfaces based on Self-assembly of Colloidal Particles

Guoxu Yu, Heyang Zhang, Yiming Li, Lele Song, Jinglin Jia, Lei Chen, Ding Weng, Yuan Ma*, Jiadao Wang*

Department of Mechanical Engineering, Tsinghua University, Beijing 100084, P.R. China

*Yuan Ma - Email: yuanma@tsinghua.edu.cn

*Jiadao Wang - Email: jdwang@mail.tsinghua.edu.cn

Guoxu Yu and Heyang Zhang contributed equally to this work and share first authorship.

**Supporting Figures**


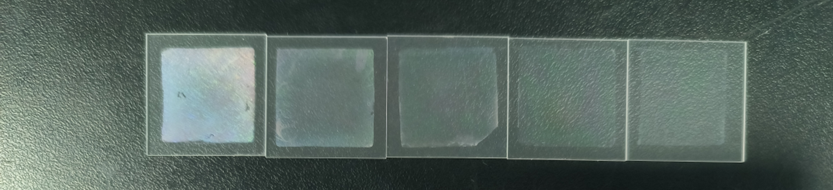


**Figure S1. The silicon glass substrate after stamping under different temperatures.** From left to right, they were 100℃, 110℃, 120℃, 130℃, and 140℃, respectively.


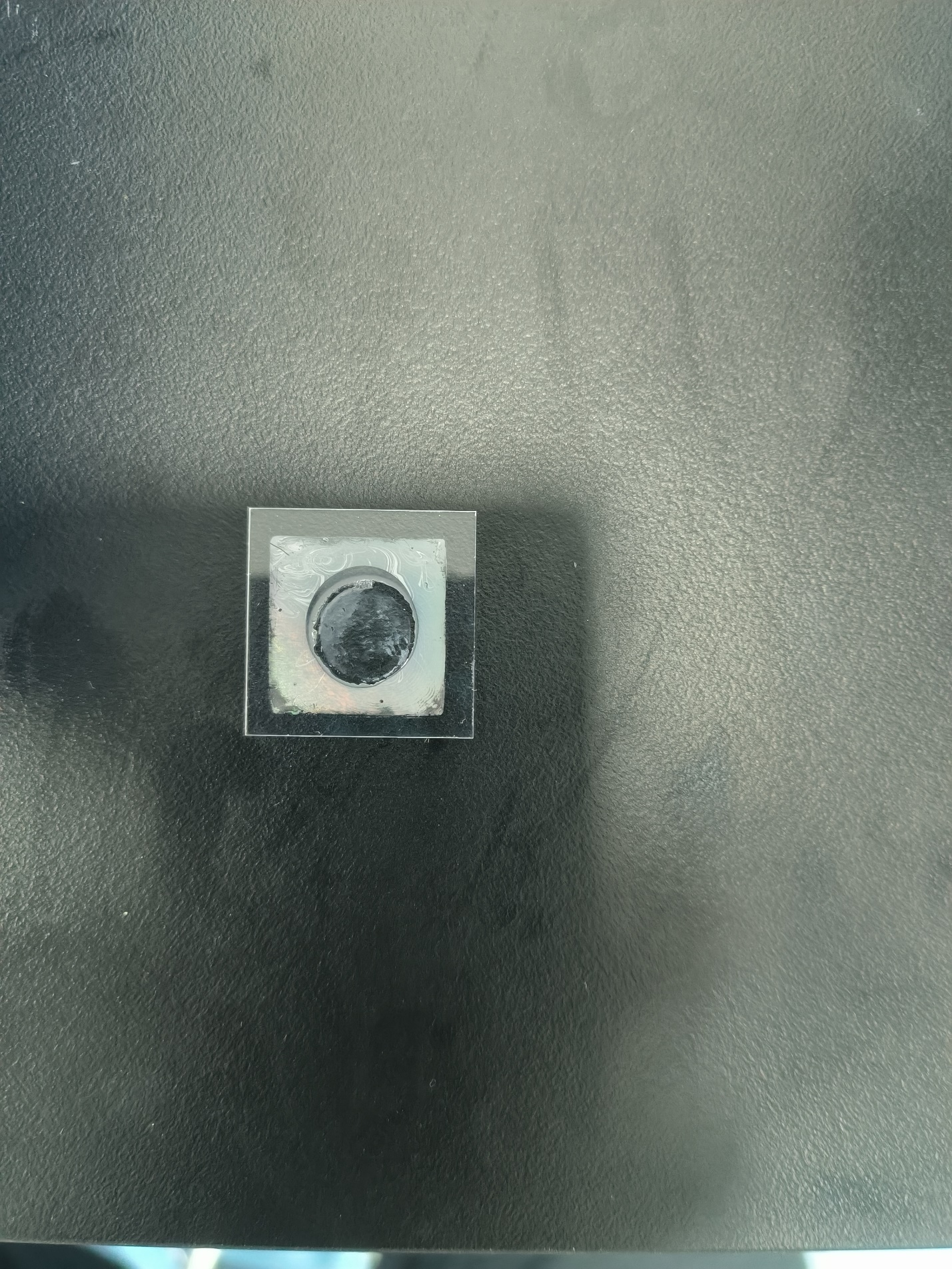


**Figure S2. The silicon glass substrate after pulling off the PS rod glued to it.**


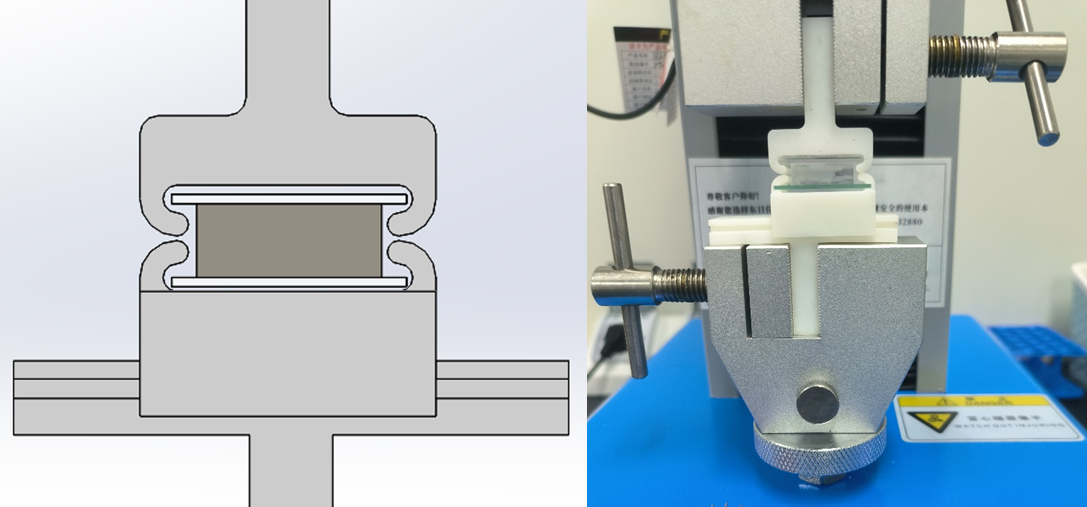


**Figure S3. The self-designed clamp for measuring bonding strength on a tensile testing machine.**


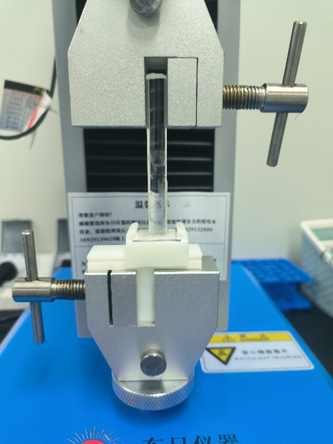


**Figure S4. Measuring bonding strength with the substrate.**


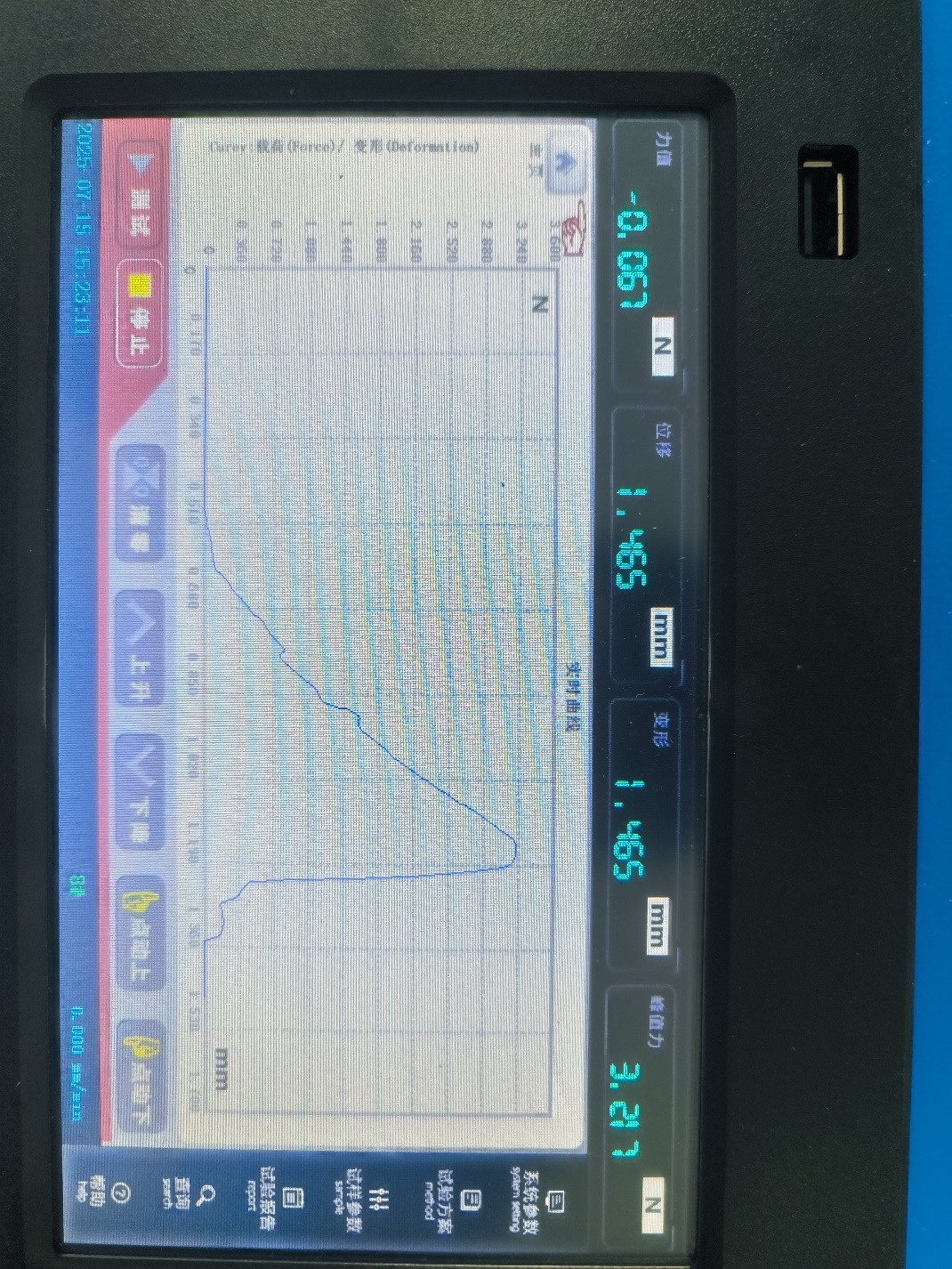


**Figure S5. One of the curves of bond strength measurement.**


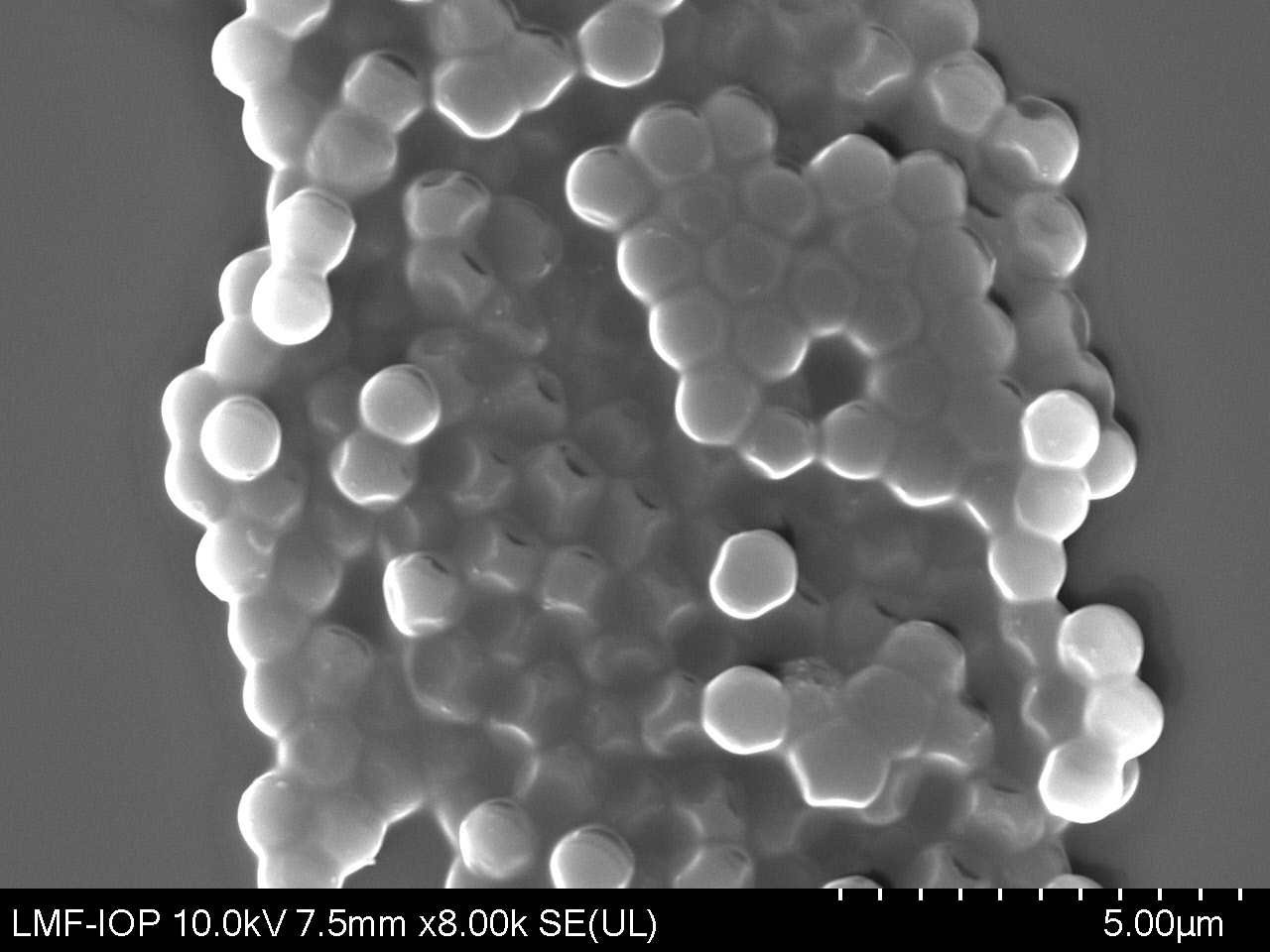


**Figure S6. The result of insufficient time during multi-layer microsphere stamping.** The insufficient transfer time resulted in an inadequate connection between the microspheres, causing them to disconnect from the middle when removing the stamp.


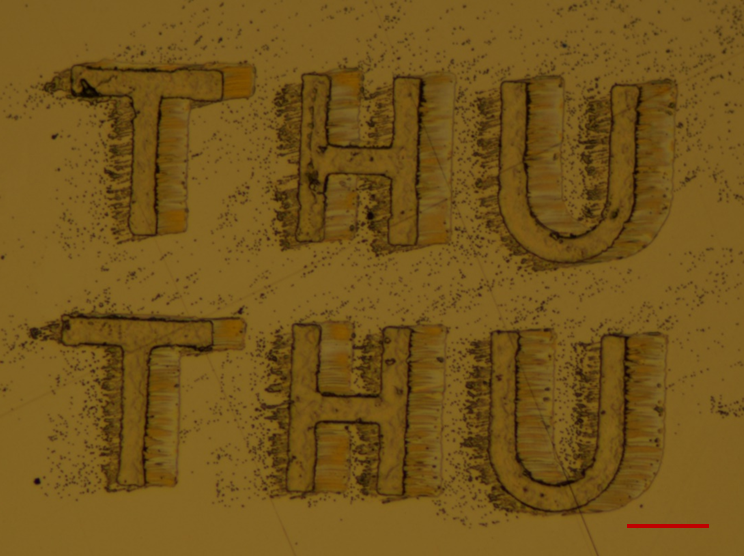


**Figure S7. The stamping result of the resist mask under 200℃ (lateral movement caused by thermal expansion)**. No external pressure was used. The pressure corresponding to this result was only the self-weight of the stamp, indicating that the PS microspheres had already shown consistent behavior with the liquid state at 200 ℃. After the stamp expanded due to heat, it moved and dragged the liquefied PS ball, leaving traces. Scale bar, 100 μm.


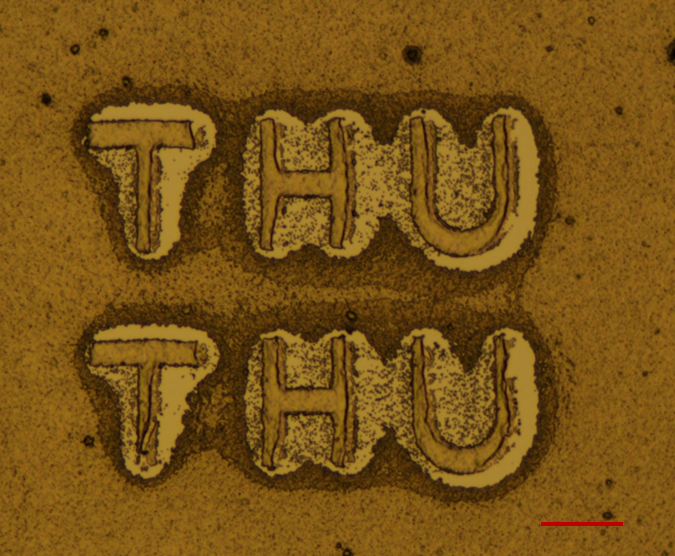


**Figure S8. The stamping result of the resist mask under excessive pressure.** Too much pressure caused excessive transfer, which meant the unwanted area was transferred as well, as shown at the edge of the letter "THU" in the picture. In addition, due to the significant deformation of the PDMS stamp, the words "THU" were also distorted and deformed. Scale bar, 100 μm.


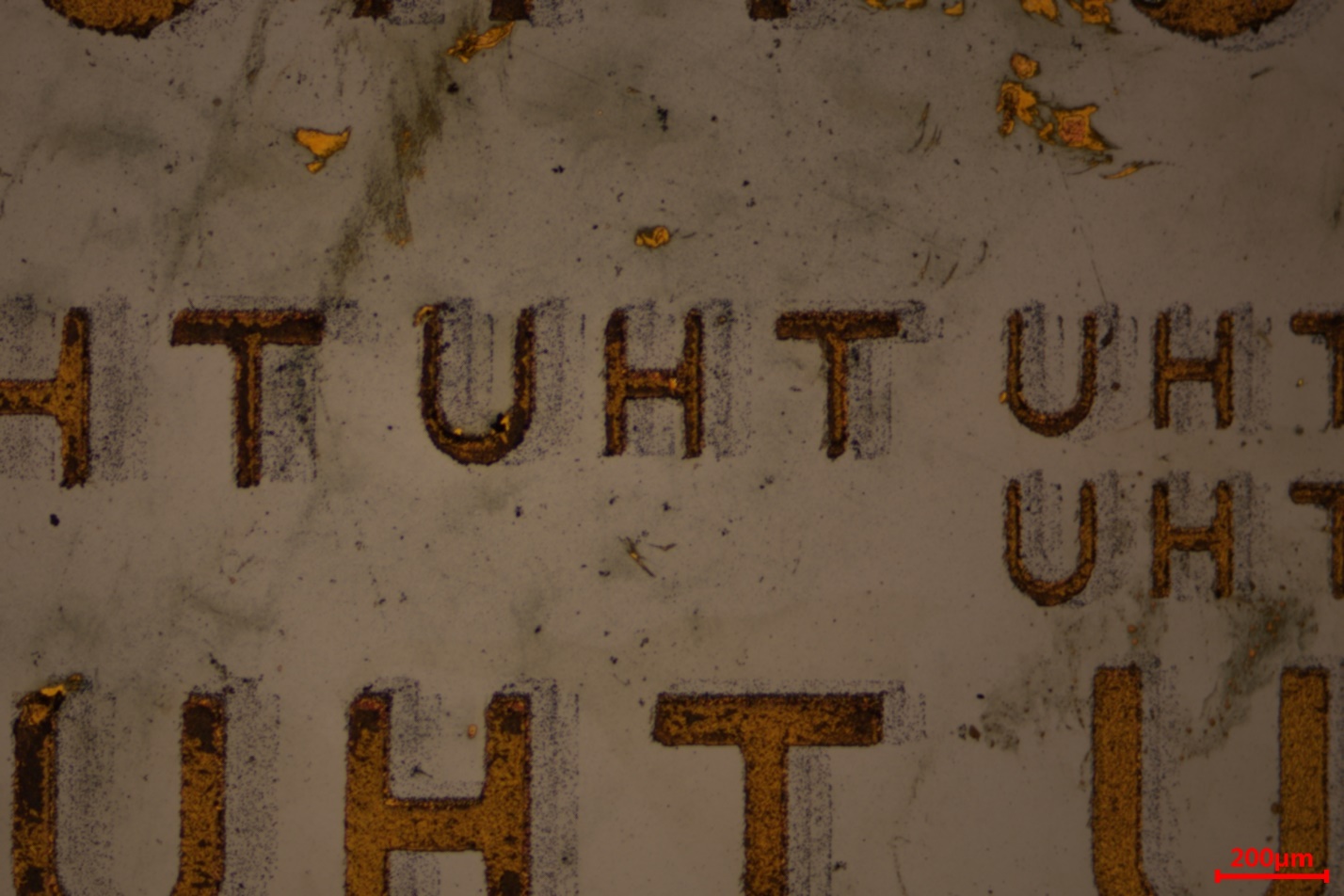


**Figure S9. The melted PS tail traces didn’t affect the morphology of the etching result.** The black parts were microspheres, which could be removed by subsequent cleaning. Scale bar, 100μm.

**
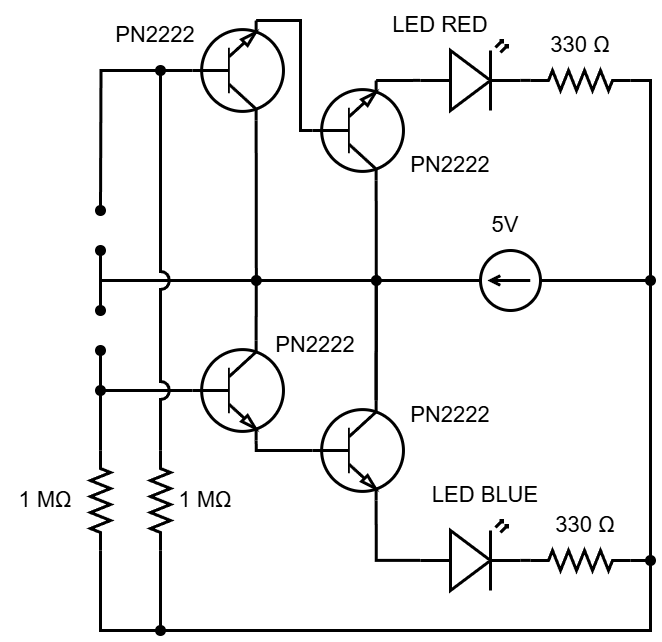
**

**Figure S10. Design of the switch circuit on the hemisphere substrate.**

**Supplementary Videos**

**Video S1**: A Touch Switch Circuit on a Glass Hemisphere
